# Using dimensionality-reduction techniques to understand the organization of psychotic symptoms in persistent psychotic illness and first episode psychosis

**DOI:** 10.1038/s41598-023-31909-w

**Published:** 2023-03-24

**Authors:** Leah M. Fleming, Ann Catherine Lemonde, David Benrimoh, James M. Gold, Jane R. Taylor, Ashok Malla, Ridha Joober, Srividya N. Iyer, Martin Lepage, Jai Shah, Philip R. Corlett

**Affiliations:** 1 Department of Psychiatry, Yale University School of Medicine, New Haven, CT USA; 2 Interdepartmental Neuroscience Department, Yale University, New Haven, CT USA; 3 Department of Psychiatry, McGill University Montreal, Qubec, Canada; 4 Maryland Psychiatric Research Center, University of Maryland School of Medicine, Baltimore, MD USA; 5 The Prevention and Early Intervention Program for Psychosis (PEPP-Montreal), Douglas Mental Health University Institute, Qubec, Canada; 6 Department of Psychology, Yale University, New Haven, CT USA; 7 Department of Neuroscience, Yale University, New Haven, CT USA; 8 Wu Tsai Institute, Yale University, New Haven, CT USA; 9 Connecticut Mental Health Center, 34 Park St, New Haven, CT 06519 USA

**Keywords:** Comorbidities, Schizophrenia

## Abstract

Psychotic disorders are highly heterogeneous. Understanding relationships between symptoms will be relevant to their underlying pathophysiology. We apply dimensionality-reduction methods across two unique samples to characterize the patterns of symptom organization. We analyzed publicly-available data from 153 participants diagnosed with schizophrenia or schizoaffective disorder (fBIRN Data Repository and the Consortium for Neuropsychiatric Phenomics), as well as 636 first-episode psychosis (FEP) participants from the Prevention and Early Intervention Program for Psychosis (PEPP-Montreal). In all participants, the Scale for the Assessment of Positive Symptoms (SAPS) and Scale for the Assessment of Negative Symptoms (SANS) were collected. Multidimensional scaling (MDS) combined with cluster analysis was applied to SAPS and SANS scores across these two groups of participants. MDS revealed relationships between items of SAPS and SANS. Our application of cluster analysis to these results identified: 1 cluster of disorganization symptoms, 2 clusters of hallucinations/delusions, and 2 SANS clusters (asocial and apathy, speech and affect). Those reality distortion items which were furthest from auditory hallucinations had very weak to no relationship with hallucination severity. Despite being at an earlier stage of illness, symptoms in FEP presentations were similarly organized. While hallucinations and delusions commonly co-occur, we found that their specific themes and content sometimes travel together and sometimes do not. This has important implications, not only for treatment, but also for research—particularly efforts to understand the neurocomputational and pathophysiological mechanism underlying delusions and hallucinations.

## Introduction

Schizophrenia is defined by five core features^1^: delusions, hallucinations, disorganized speech, grossly disorganized/catatonic behavior and negative symptoms. To be diagnosed, a person must exhibit at least 2 of these with at least one being delusions, hallucinations or disorganized speech. This means that two individuals can be diagnosed with schizophrenia despite having no symptoms in common.

Because of this heterogeneity, many scientists have adopted a symptom rather than syndrome approach. They study the mechanisms of delusions while attempting to control for other symptoms^2^. Such focus is common in cognitive neuropsychiatry^3^ and more recently computational psychiatry^4^. Since delusions and hallucinations often co-occur, and respond to drugs that block dopamine D_2_ receptors, some accounts—like Kapur’s aberrant incentive salience theory^5^, attempt to explain both hallucinations and delusions within the same framework. Such efforts while admirable, have not withstood empirical examination. Hallucinations and delusions emerge separately^6^ and resolve differently with treatment^7,8^. Models and data that explain delusions often appeal to orthogonal mechanisms to those invoked for hallucinations^9–11^. However, some accounts suggest attending to symptom themes may help; commonality across passivity delusions (beliefs that one’s actions or thoughts are under the control of an external agent) and auditory verbal hallucinations exists^12^.

Thus, conclusions about the biological and computational basis of psychosis are impacted by how these symptoms co-occur. We believe that the clinical phenomenology should guide our construction of explanatory models^13^. However, we favor data driven rather than descriptive approaches to the organization of psychotic symptoms. The overarching aim of this work is to enrich and constrain accounts of positive symptoms which span biology and behavior by studying the ways in which positive symptoms co-occur.

A growing literature employs data-driven approaches to clarify relationships among psychotic symptoms^14–17^ and has been shown to predict important long-term outcomes better than conceptual distinctions (e.g. positive and negative symptoms)^18^. However, these studies have used different techniques, in varying samples which sometimes conflate phases of illness, diagnoses, and medication experience^17^. For instance, Minas and colleagues^19^ applied multidimensional scaling (MDS) to the Scale for the Assessment of Positive Symptoms (SAPS) and the Scale for the Assessment of Negative Symptoms (SANS) scores in patients with various psychotic illnesses. Others have used principal component analysis (PCA) with global symptoms^20^, or various subsets of reality distortion symptoms in studies of patients with schizophrenia^14^ or in FEP samples^15,16^. And others still have conducted exploratory factor analysis techniques^18,20–22^. There are consistent findings: a three factor division is apparent^23^ (Reality distortion, Disorganization, Negative Symptoms), but subdivisions within these also likely exist^17,18,20,21^, and when reality distortion is examined more closely, specific delusional contents were more related to auditory hallucinations (e.g. passivity delusions) while other delusion themes (such as grandiose delusions) were not^14–16,19,23^. However, there remains many inconsistencies across these studies, leading to varying conclusions about symptom organization.

We therefore sought to replicate and extend these findings in publicly available SAPS and SANS measures from patients with persistent psychotic illness, applying MDS and cluster analysis to study latent symptom structure. MDS can characterize and visualize the overall structure of how symptoms relate to one another. This method is optimal for visualizing relationships between items^24^. Non-metric MDS is especially suitable for ordinal data like SAPS and SANS^19^. Similar methods have been used previously^19^, but advances in statistical techniques can increase confidence and interpretation. Our focus is on understanding how different types of hallucinations and delusions relate to each other, but we ground these analyses by also including the two other hallmark categories of symptoms experienced by patients: thought disorder (from SAPS) and negative symptoms (from SANS). Using MDS, we can thus visualize the relationships amongst our items of interest and also their proximity to other important symptoms. This can also be achieved with PCA, which we report in the Supplement.

We also repeated our analyses on an independent, minimally medicated FEP sample, and further separated this sample by diagnostic subtype (affective v. nonaffective psychosis), to examine the consistency of symptom organization across illness chronicity, medication exposure and psychotic illness sub-type.

Consistent with previous findings^14–16,19^ we hypothesized that MDS would yield a three-factor division: reality distortion, thought disorder and negative symptoms^23^, but would also help identify and clarify further delineations of reality distortion.

In summary, we sought to study the inter-relationships between hallucinations and delusions (with different contents). We hope that the outcome of our efforts may assist computational psychiatry approaches to psychotic symptoms, which have begun to identify common and distinct processes in delusions and hallucinations.

## Results

### Persistent psychotic illness sample

**Figure 1:**
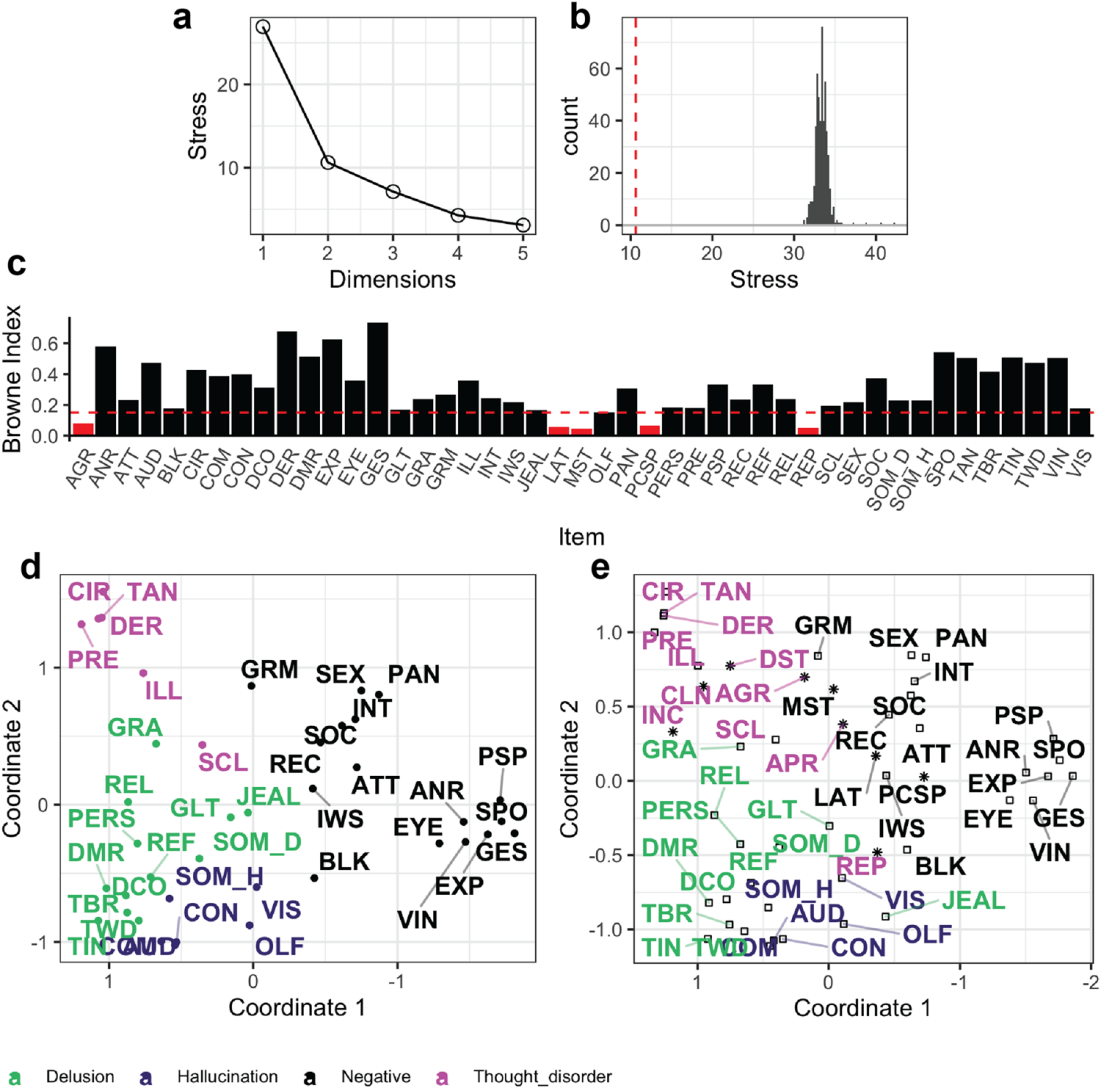
Multidimensional scaling solution for persistent psychotic illness sample. (**a**) Scree plot representing stress on the model across different potential numbers of dimensions used in the solution. (**b**) Permutation test results with distribution of estimated stress from 500 randomly permuted sets of our data. Stress in the experimental solution (red line) was significantly less (p < 0.001). (**c**) Communality exclusion criteria shown based on Browne index values plotted across all items of SAPS and SANS. Cutoff criteria indicated by red dashed line; those items lying below this criterion and therefore excluded filled in red (items which failed to pass inclusion criteria for endorsement not shown). (**d**) Two-dimensional solutions for our MDS analysis. The colors are added to differentiate subgroups of symptoms previously identified in the literature. (**e**) MDS solution with all items included (no exclusion criteria) with items that met inclusion criteria labeled by the box and items which were excluded from the original analysis (and therefore not included in **d**) in asterisk. CON: voices conversing, COM: voices commenting, SOM_H: somatic hallucinations, VIS: visual hallucinations, OLF: olfactory hallucinations, SOM_D: somatic delusions, GRA: grandiose delusions, REL: religious delusions, PERS: persecutory delusions, REF: delusions of reference, DCO: delusions of being controlled, GLT: delusions of guilt/sin, JEAL: delusions of jealousy, DMR: delusions of mind reading, SCL: social and sexual behavior, TAN: tangentiality, TBR: thought broadcasting, DER: derailment, TWD: thought withdrawal, INC: incoherence, ILL: illogicality, CIR: circumstantiality, PRE: pressure of speech, DST: distractible speech, TIN: thought insertion, GRM: grooming and hygiene, EXP: unchanging facial expression, SPO: less spontaneous activity, GES: paucity of gestures, EYE: poor eye contact, ANR: affective non-responsiveness, VIN: low vocal inflection, PSP: poverty of speech, BLK: blocking, IWS: impersistence at work, PAN: physical anergia, SEX: sexual activity, SOC: relationships with friends, INT: Inability to feel intimacy, REP: repetitive and stereotyped behavior, AGR: aggressive and agitated behavior, LAT: increased latency of response, CLN: clanging, APR: clothing and appearance, MST: inattentiveness during mental status testing, PCSP: poverty of content of speech.

Four SAPS items were excluded from the MDS analysis because of low endorsement and an additional two SAPS and three SANS items excluded for low communality (Table S1; Fig. 1c).

A 2-dimensional solution was selected because there was a large reduction in stress between 1 and 2 dimensions, but little reduction for subsequent dimensions (Fig. 1a). Stress is a goodness-of-fit metric of the difference between the observed similarity matrix and the estimated pairwise distance matrix. Our model had significantly lower stress than 500 random iterations (p < 0.001) (Fig. 1b). The MDS solution (Fig. 1d) had an R^2^-value of 0.92, indicating a strong relationship between items in this 2D space and their pairwise correlation coefficients.

To ensure our results were not biased by our inclusion criteria, we re-ran the MDS with all items of SAPS and SANS (Fig. 1e). The R^2^ value for this solution was lower at 0.903 and the stress on the model increased from 10.62 to 11.87. Additionally, when we apply a Procrustes transformation across the two models, we find that the congruence for the two dimensions is very high at 0.99 and 0.97 respectively. This confirms our exclusion criteria did not influence the solution extensively. We next re-ran our MDS with only SAPS items which met our inclusion criteria, to determine whether inclusion of SANS affects our results (Fig. S1). The localization of SAPS items was consistent when SANS were excluded versus included, with congruence between the two sets of vertical and horizontal dimensions at 0.98, and 0.76 respectively.

**Figure 2:**
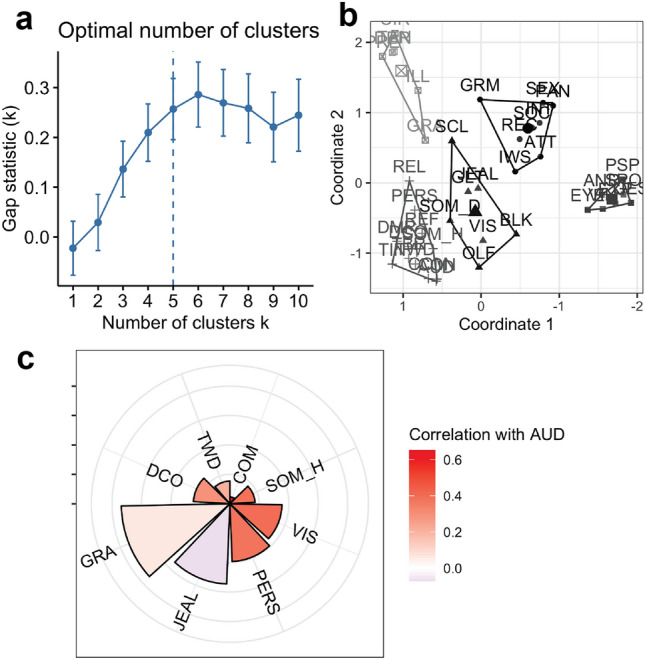
MDS follow-up cluster analysis and relationship with correlation coefficients. (**a**) Gap statistics across different number of clusters for k-means cluster analysis of persistent illness sample. Optimal number of clusters marked with vertical dashed line. (**b**) K-means cluster analysis solution with 5 clusters. (**c**) Relative distances between various reality distortion items and auditory hallucinations (AUD) from MDS with their pairwise correlation coefficients in color. CON: voices conversing, COM: voices commenting, SOM_H: somatic hallucinations, VIS: visual hallucinations, OLF: olfactory hallucinations, SOM_D: somatic delusions, GRA: grandiose delusions, REL: religious delusions, PERS: persecutory delusions, REF: delusions of reference, DCO: delusions of being controlled, GLT: delusions of guilt/sin, JEAL: delusions of jealousy, DMR: delusions of mind reading, SCL: social and sexual behavior, TAN: tangentiality, TBR: thought broadcasting, DER: derailment, TWD: thought withdrawal, INC: incoherence, ILL: illogicality, CIR: circumstantiality, PRE: pressure of speech, DST: distractible speech, TIN: thought insertion, GRM: grooming and hygiene, EXP: unchanging facial expression, SPO: less spontaneous activity, GES: paucity of gestures, EYE: poor eye contact, ANR: affective non-responsiveness, VIN: low vocal inflection, PSP: poverty of speech, BLK: blocking, IWS: impersistence at work, PAN: physical anergia, SEX: sexual activity, SOC: relationships with friends, INT: Inability to feel intimacy.

We performed a k-means cluster analysis with the optimal number of clusters based on gap statistic (Fig. 2a,b). Voice-hearing symptoms clustered with passivity delusions, while delusions of reference and persecution (paranoid) also localized towards the upper bound of this cluster. Next, we identified a cluster containing non-auditory hallucinations with all other types of delusions. Another cluster comprised all items from the formal thought disorder section of SAPS and grandiose delusions. The remaining SANS items were divided into two distinct clusters: asocial and apathy versus speech and affect.

Figure 2c shows the relative distances from auditory hallucinations (AUD) to a range of key reality distortion symptoms in the MDS solution, color-weighted by their bivariate correlation with AUD. Voices communicating are almost coincident and highly correlated, whereas visual hallucinations are further and weaker, as are persecutory delusions, grandiose delusions and delusions of jealousy. To further ensure that our results were not driven by unrelated characteristics of the symptom items like overall endorsement or differing variance in reported scores, we computed the correlation between distance from AUD and mean item rating across all participants (r = 0.17, p = 0.30) as well as standard deviation in item rating (r = − 0.01, p = 0.97) and found no relationship with either.

### First episode psychosis (FEP) vs persistent illness

**Figure 3:**
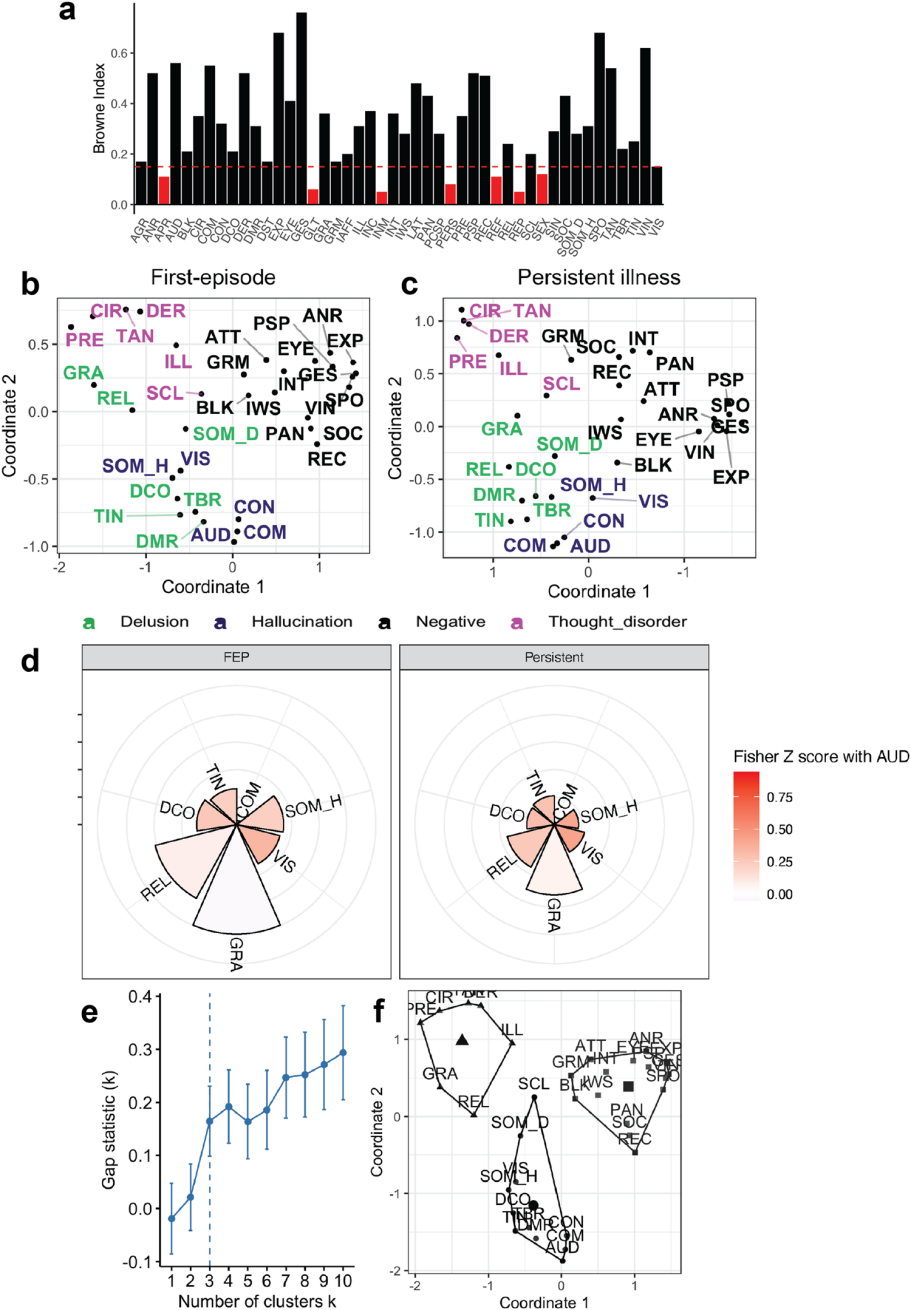
MDS solution for first-episode. (**a**) Browne index values for items from the FEP sample. Items in red are below threshold for inclusion. (**b**) MDS solution for FEP and (**c**) persistent illness sample with items which met inclusion at both sites. (**d**) Relative distances between various reality distortion items and auditory hallucinations (AUD) from MDS with their pairwise Fisher’s Z transformed correlation coefficients in color for FEP and persistent illness samples compared side-by-side. (**e**) Gap statistics across different number of clusters for k-means cluster analysis of FEP sample. Optimal number of clusters marked with vertical dashed line. (**f**) K-means cluster analysis solution with 3 clusters. CON: voices conversing, COM: voices commenting, SOM_H: somatic hallucinations, VIS: visual hallucinations, OLF: olfactory hallucinations, SOM_D: somatic delusions, GRA: grandiose delusions, REL: religious delusions, PERS: persecutory delusions, REF: delusions of reference, DCO: delusions of being controlled, GLT: delusions of guilt/sin, JEAL: delusions of jealousy, DMR: delusions of mind reading, SCL: social and sexual behavior, TAN: tangentiality, TBR: thought broadcasting, DER: derailment, TWD: thought withdrawal, INC: incoherence, ILL: illogicality, CIR: circumstantiality, PRE: pressure of speech, DST: distractible speech, TIN: thought insertion, GRM: grooming and hygiene, EXP: unchanging facial expression, SPO: less spontaneous activity, GES: paucity of gestures, EYE: poor eye contact, ANR: affective non-responsiveness, VIN: low vocal inflection, PSP: poverty of speech, BLK: blocking, IWS: impersistence at work, PAN: physical anergia, SEX: sexual activity, SOC: relationships with friends, INT: Inability to feel intimacy.

The first-episode psychosis sample had very similar patterns of symptom organization as compared to the persistent illness group (Fig. 3b,c). The items that did not meet criteria for inclusion for MDS analysis in this dataset were different from the original dataset (Table S1; Fig. 3a). Therefore, items that did not meet the criteria for *both* samples were removed, and MDS was run on the remaining items in this sample and re-run in the original sample, producing solutions with R^2^-values of 0.93 and 0.96 respectively. Again, passivity delusions and auditory-type hallucinations localized together, then grandiose and religious delusions localized together, closer to thought disorder symptoms (Fig. 3b). The distance from auditory hallucinations and respective Fisher’s Z transformed correlation coefficient increased from voices commenting and passivity delusions to visual hallucinations and grandiose delusions consistently across both samples (Fig. 3d).

Note that because more items were excluded to accommodate comparison across datasets, patterns of localization for negative affect and paranoid delusions could not be observed, but the general organization was consistent. The MDS solutions across persistent and first-episode samples had high congruence coefficients, 0.941 and 0.864 for the first and second dimension respectively.

The cluster analysis for the FEP sample revealed only three clusters as the optimal solution according to gap statistics (Fig. 3e). The solution generally depicts a negative symptom cluster, a reality distortion cluster and a thought disorder cluster, although delusions separate into different clusters with grandiose and religious delusions clustering with thought disorder symptoms (Fig. 3f).

We next computed Fisher’s z-transformed r for each correlation with auditory hallucinations within each dataset and tested for statistical differences applying Bonferroni correction for multiple comparisons. The within-cluster correlations with auditory hallucinations were significantly stronger than those with items that fell outside the auditory hallucinations cluster. We also found that the Bonferroni corrected strength of between symptom correlations with auditory hallucinations did not differ between the datasets (Table S3).

### Affective FEP vs non-affective FEP

**Figure 4:**
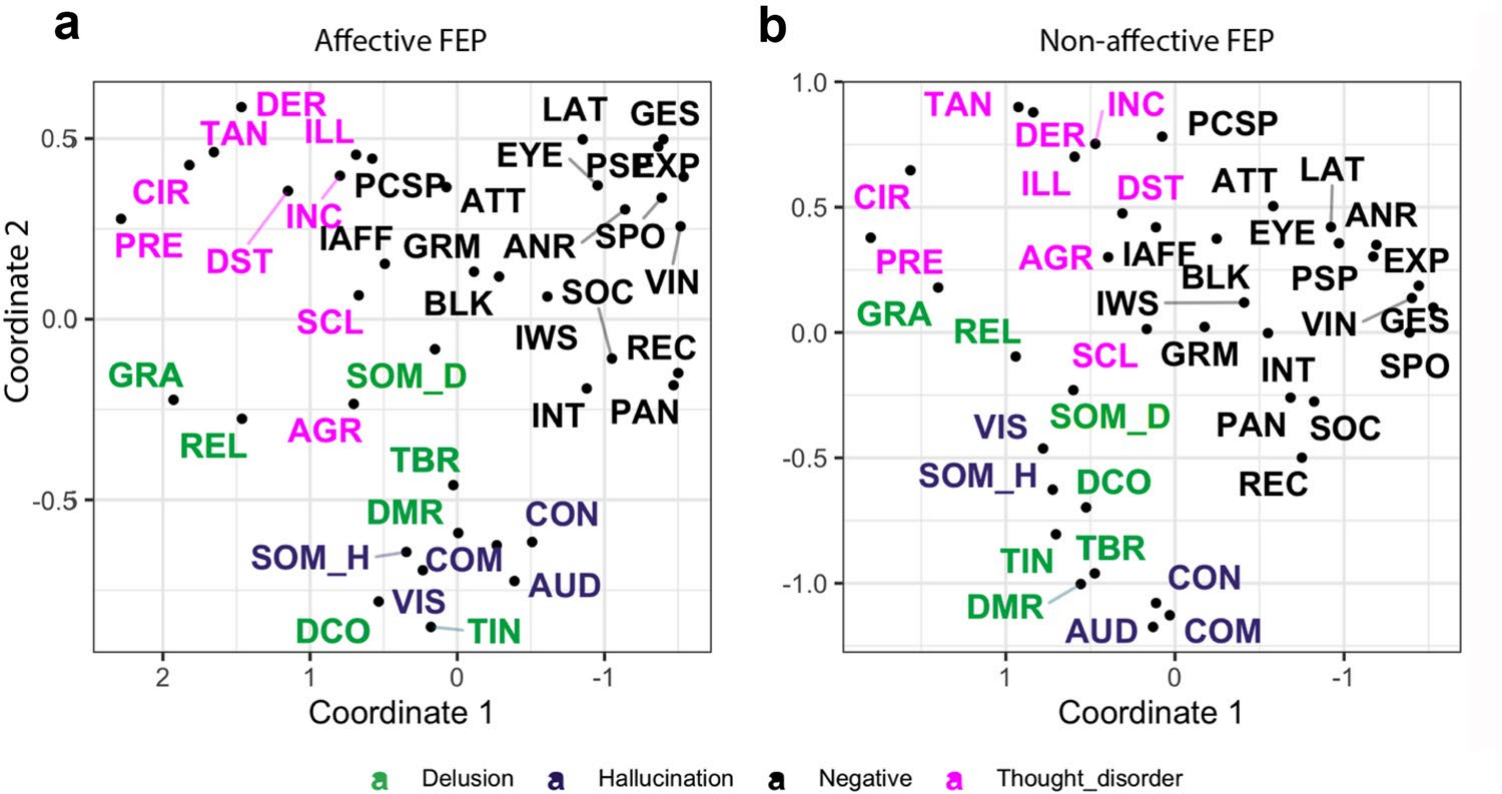
MDS solution for subgroups of FEP sample. (**a**) MDS solution for participants with an Affective FEP and (**b**) Non-affective FEP diagnosis. CON: voices conversing, COM: voices commenting, SOM_H: somatic hallucinations, VIS: visual hallucinations, OLF: olfactory hallucinations, SOM_D: somatic delusions, GRA: grandiose delusions, REL: religious delusions, PERS: persecutory delusions, REF: delusions of reference, DCO: delusions of being controlled, GLT: delusions of guilt/sin, JEAL: delusions of jealousy, DMR: delusions of mind reading, SCL: social and sexual behavior, TAN: tangentiality, TBR: thought broadcasting, DER: derailment, TWD: thought withdrawal, INC: incoherence, ILL: illogicality, CIR: circumstantiality, PRE: pressure of speech, DST: distractible speech, TIN: thought insertion, GRM: grooming and hygiene, EXP: unchanging facial expression, SPO: less spontaneous activity, GES: paucity of gestures, EYE: poor eye contact, ANR: affective non-responsiveness, VIN: low vocal inflection, PSP: poverty of speech, BLK: blocking, IWS: impersistence at work, PAN: physical anergia, SEX: sexual activity, SOC: relationships with friends, INT: Inability to feel intimacy.

When the FEP sample was split into affective (Fig. 4a) and non-affective (Fig. 4b) psychosis, we observed a similar spatial organization of symptoms in both subgroups. After Procrustes transformations, the congruence coefficients between the first and second dimensions were 0.960 and 0.933 respectively.

We further tested whether reality distortion symptoms that cluster together in the MDS solution load similarly in a principal component analysis and did find distinct groupings of symptoms which load together in PCA and are also more closely aligned in MDS for both persistent illness (Fig. S2) and FEP (Fig. S3).

To draw further comparisons between the two datasets (as we have with the Procrustes transformations, congruence analysis and PCA) we sought to demonstrate measurement invariance (of the SAPS and SANS) between the two datasets. To do so, we first conducted an exploratory factor analysis on the FEP sample (the larger of our two datasets) to determine the optimal number of factors and which items make up each factor. Based on a minimized BIC and on visualizing the elbow of the scree plot (Fig. S4), a 5-factor solution was the best model.

We then input this 5-factor model to a multigroup confirmatory factor analysis (m-CFA)—including both the FEP and Persistent Illness datasets—to test for measurement invariance using established methods^25^ across the two samples, excluding participants with missing data. These methods were implemented in R using the lavaan and semtools packages.

We start by determining whether a consistent factorial structure can be fit across our groups with a configural invariance model using a WLSMV estimator. The model fit our groups well (RMSEA = 0.032) based on established cutoff criteria for goodness of fit indices^25^.

Next, we tested for metric invariance by fixing the factor loadings across the FEP and persistently ill groups to be equivalent and find this model has a good fit and is not statistically different from the original model (χ^2^ = 32.5, df = 27, p = 0.21, RMSE = 0.028).

Since we established metric invariance, we next explored whether there was scalar invariance by setting both the loadings and intercepts equal across our groups. Despite a good fit for this model, we do see a significant difference between this new, more constrained model and the previous model (χ^2^ = 1103.1, df = 27, p < 0.01, RMSEA = 0.029) so we ease our constraints and allow some items’ intercepts to be freely estimated across groups^26^.

The items which were allowed to vary freely were: PRE, TBR, SOM_H, AGR, PSP, EYE, CON, GRA, REL, IWS and GRM. The chi-square difference test between this partially fixed model and our original model was not statistically different with a similar model fit ((χ^2^ = − 16.2, df = 14, p > 0.9, RMSEA = 0.028), indicating partial metric invariance between the groups on these SAPS and SANS items.

Finally, we tested for the most stringent measurement invariance, strict invariance, where loadings, intercepts and residuals are fixed to be the same across our groups, allowing the same items to have freely estimated intercepts as in the previous model. We were not able to establish strict invariance because this model was statistically different from our previous model (χ^2^ = 91.5, df = 32, p < 0.01, RMSEA = 0.028).

Taken together, we suggest there is configural and metric invariance on the SAPS and SANS across the two datasets, as well as good partial scalar invariance. However, our datasets lack strict invariance.

Establishing configural and metric invariance is the most important for our purposes. Configural invariance tells us that the items load in the same direction across the two datasets. Metric invariance tells us that each item loads onto each factor (latent variable) by the same amount (i.e. the slope between the item and the factor are equal) across the two datasets. Hence, a change from a 1 to a 2 is the same in FEP and in chronically ill samples.

We fell short of demonstrating strict invariance. Strict invariance suggests that the explained variance for every item is the same across groups. Put more strongly, the latent construct is measured identically across groups. We cannot claim identity. However, based on the Procrustes analysis, and the other invariance analyses, we feel confident in claiming that the organization of positive symptoms is similar in first episode and chronic schizophrenia. We were most interested in whether the organization of symptoms is consistent, not whether the magnitude of endorsement of each cluster/factor is the same across our groups, so we did not compare the magnitude of endorsement of each symptom or each factor in each dataset. Our analyses suggest similarities in the inter-relationships between hallucinations and delusions across two independent samples from different illness phases. Since we did not establish strict measurement invariance, we cannot conclude that these patterns are identical across datasets, but they are highly similar.

## Discussion

In order to further understand how symptoms of psychotic illnesses relate to each other, we leveraged data-driven approaches^14–16,19,27^ in independent persistent and early-stage clinical samples. We used dimensionality-reduction techniques in parallel across samples, generating a robust and replicable relationship and distinction of symptoms. Our two samples differ in age, length of illness, diagnoses and experience with antipsychotic medication, yet the organization of MDS and the loadings of PCA were highly related across samples. This indicates that symptom organization is conserved across illness phase and across confounds like medication exposure and diagnosis.

Many of the scales and diagnostic tools used to study psychotic symptoms (including SAPS and SANS) currently combine items into conceptual categories. However, our results challenge the idea that hallucinations versus delusions, for instance, are the best categories for organizing symptom research. Our data-driven approaches suggest instead studying symptoms which are statistically rather than conceptually related. In fact, recent neuroimaging research has shown that PCA components of symptom scales were superior for identifying robust correlates of brain connectivity compared to traditional scale sub-scores^28^.

Previous work has focused on EFA and PCA to determine how many factors best capture symptom dimensions. Varying conclusions have been drawn, and in many cases a handful of symptoms do not fit into any factor^18^. Our MDS results can illuminate the relationships between items and give a more fine-grained understanding. Our results in the persistently ill sample capture both the overall distinction of reality distortion from other psychotic symptoms generally, and the clear pattern of separation of auditory hallucinations and passivity delusions from grandiose and negative affect delusions as well as non-auditory hallucinations. We can even draw conclusions at the level of individual symptoms within clusters, especially given the strong replication across our samples. And in both samples, we find that reality distortion items which are further from AUD have low to zero correlation coefficients with AUD (Fig. 3d), suggesting divergent underlying pathophysiological mechanisms.

One key difference between our persistently ill and FEP samples we observed was which items were excluded due to low endorsement or low communality with other items (Table S1). Further work could directly determine whether there are meaningful differences in these items which differ on our inclusion criteria. Importantly amongst those symptoms which do meet both inclusion criteria, congruence is high and the relationship between auditory hallucinations and other reality distortion symptoms is maintained (Fig. 3d).

Our results overall are consistent with many previous studies showing divisions in reality distortion symptoms exist^14–16,19,27^; even Liddle’s original factor analysis work indicated a division amongst reality distortion^23^. He reported a group of reality disintegration symptoms (mostly Schneiderian first rank symptoms like auditory verbal hallucinations and passivity delusions) and a group of what Liddle termed integrative reality distortion symptoms (like persecutory, referential, and grandiose delusions). This sub-structure within reality distortion did not become a focus for future research. We are delighted to replicate and extend it presently.

We show here a symptom-level visualization of these divisions amongst reality distortion. This symptom structure could aid in elucidating the underlying biopsychosocial causes of symptoms, informing and improving clinical practice. Dense-array EEG has revealed differences in cortical high gamma oscillations in patients with passivity delusions and auditory hallucinations compared to those lacking such symptoms^29^. These separate symptom clusters may also predict different treatment response and prognosis better than traditional diagnoses^30^. In fact, delusion severity and hallucination severity at symptom onset was shown to predict different clinical outcomes^31^; specific delusion and hallucination clusters may be most predictive of these outcomes.

This framework could also inform our computational understanding of reality monitoring. Hierarchical perceptual-inference models have been proposed to help explain both commonalities and differences related to hallucinations and delusions^9,10^; our work suggests such accounts must recognize that some delusions occur with hallucinations, and may share mechanisms, while others do not. Auditory hallucinations and passivity delusions may be driven by alterations at lower levels of an inferential neural hierarchy (more proximal to sensory inputs)^10,32^. Other delusions, conversely, may be driven by changes in processing at higher (more abstract) levels of these hierarchical models^9,10^. For instance, paranoia has been connected to prior beliefs about how contingencies might shift dynamically^22,33^ and grandiosity to more severe cognitive reasoning biases^34^. Phenomenologically, our vertical axis may represent this spectrum from higher-order, more cognitive deficits grounded by disordered thought at the top, moving through delusions of grandiosity and persecution^35^ to lower-order, more perceptual deficits anchored by auditory hallucinations^34,36^. While this cannot be formally tested here, our hypothesis would predict that parameters of hierarchical perceptual-inference models which related to higher levels of the hierarchy (and activity in brain regions proposed to anchor these levels) are more related to certain delusions and thought disorder, while parameters at lower levels of the model should relate more to passivity delusions and hallucinations.

## Strengths and limitations

Symptom severity does change with illness progression in schizophrenia^37^ and negative symptoms severity, in particular, can be unstable and challenging to measure over the course of first-episode psychosis^38^. We do observe separation between the groups in the differences in exclusion criteria across samples and this may also drive the difference in the cluster analysis results in our FEP sample with additional delusion and hallucination items excluded. However, despite these known differences across illness stage, the *organization* of symptoms is highly consistent across our two samples, supporting the idea that this symptom organization is conserved across illness phases.

Our MDS analyses used the SAPS and SANS because these scales provide detailed symptom information and generally have high temporal stability, IRR^39^ and construct validity^40^. We do lack IRRs for the persistent psychotic illness sample here, which was collected across multiple sites, and the FEP sample was collected over 15 years across multiple raters without complete IRRs for each rater, which could introduce additional variability. However, this makes it even more remarkable that these results show such high consistency across samples and subsamples, with this additional introduction of variance across participants.

It is also possible some patterns we observe could be driven by characteristics of these particular scales we chose. Although we focus on relationships amongst positive symptoms, we do include items of SANS. The SANS includes several items which are considered secondary to other symptom and thus not true negative symptoms (e.g. attention disturbance, poverty of content of speech, increased latency to response, inappropriate affect)^41,42^; most of these items were excluded because of low endorsement or communality (Table S1) which supports the argument that these represent a different construct from negative symptoms. In the chronically-ill sample, our results are consistent with the conclusion that SANS is composed of two separatable components, which is consistent with previous results using factor analysis and identifying separable motivational versus diminished expression groups of symptoms^43,44^. Importantly, because we applied MDS across both SAPS and SANS, we show here that neither of these clusters of SANS items overlap strongly with SAPS items either, demonstrating they offer unique contributions. Also, we show that the organization of SAPS items is generally consistent regardless of whether we include SANS or not (Fig. S1) so our conclusions about reality distortion symptoms stand with or without the SANS.

## Future direction

Future studies can explicitly compare symptom organizations across medicated and unmedicated patients, or in relation to length of medication exposure. Our results across samples indicate that medication status is unlikely to drive symptom relationships observed.

Furthermore, more future work might apply similar techniques longitudinally and in at-risk youth to determine whether early-stage symptoms predict later manifestations, or indeed conversion to psychosis. Previous work already supports the hypothesis that specific types of symptoms and their order of onset can predict outcome^6,18,45^.

Finally, methods for visualizing dimensional structure in data are developing rapidly^46^. As we replicate and extend these observations we will employ those new methods, which may reveal further relationships amongst symptoms.

## Conclusions

We found that certain symptoms of psychosis co-occur whilst others do not, and this is relatively consistent across samples of patients at different illness phases. Like the report from Peter Liddle in 1987 (in a small sample of patients with chronic schizophrenia) we identified two reality distortion clusters, one disorganization cluster and two SANS clusters (asocial and apathy, and speech and affect). One reality distortion cluster contained auditory verbal hallucinations as well as passivity delusions. The other reality distortion cluster contained religious and referential delusions and non-auditory hallucinations, while grandiose delusions clustered separately with disorganization items. Perhaps these clusters are underwritten by different neurocomputational mechanisms, which may portend different prognoses and treatment responses. Leveraging these relationships for clinical practice and research will deepen our understanding of psychosis.

## Methods

### Participants

#### Persistent psychotic illness sample and scales

Clinical measures from 153 participants with a persistent psychotic disorder were obtained from open sources (see Supplementary Methods for more details on participants). The SAPS and SANS, two structured, patient-interview, 6-point rating scales developed specifically for schizophrenia, were administered to participants. These scales are consistent with other measures of positive and negative symptoms^47^. We included all items and determined inclusion for statistical testing in a data-driven manner (see “Statistical methods”).

#### FEP sample

We accessed a sample of 636 participants experiencing first-episode psychosis from the Prevention and Early Intervention Program for Psychosis (PEPP-Montreal). All participants had a diagnosis of affective or non-affective psychotic illness according to SCID-IV and received antipsychotic medications for no more than 30 days upon entry (average at the date of assessment was 21.7 days). They were administered the SAPS and SANS by trained raters^48^ with moderate to high interrater reliability (IRR)^49^. Clinical assessments took place within 1-month of first intake into the PEPP-Montreal clinical services program and participants were asked to recall their most acute state from the last 3 months during assessment. Demographic and clinical information can be found in Table 1.

**Table 1:** Demographic and clinical measures in persistent psychotic illness participants divided by open-access data source (fBIRN or UCLA), as well as the FEP replication sample.

Demographics	fBIRN n (%)	UCLA n (%)	Statistics^a^	PEPP-Montreal n (%)	Statistics^b^
Sex			χ^2^ = 0.35, p = 0.55		χ^2^ = 0.47, p = 0.79
Female	31 (30%)	12 (24%)		191(30%)	
Male	72(70%)	38 (76%)		444(69.8)	
Missing	0	0 (0%)		1 (0.2%)	
Age			T = 0.42, p = 0.68		T = 15.1, p < 0.001
Mean (SD)	37.2 (11.3)	36.5(8.9)		23.8(4.75)	
Range	19–61	22–49		14–35	
Education level			χ^2^ = 4.55, p = 0.10		χ^2^ = 25.4, p < 0.001
Completed HS (or 12+ grades)	79 (76.7%)	38 (76%)		405 (63.7%)	
Did not complete HS (or < 12 grades)	9 (8.7%)	9 (18%)		194 (30.5%)	
Missing/not specified	15 (14.6%)	3 (6%)		37 (5.9%)	
Visible minority status			χ^2^ = 31.0, p < 0.001		χ^2^ = 69.0, p < 0.001
White	73 (71%)	33 (66%)		374 (58.8%)	
Black	19 (18%)	2 (4%)		83 (13.1%)	
Asian	2 (2%)	1 (2%)		49 (7.7%)	
Aboriginal	0 (0%)	11 (22%)		2 (0.3%)	
More than 1	0 (0%)	1 (2%)		NR	
Other	0 (0%)	0 (0%)		87 (13.7%)	
Missing/not specified	9 (9%)	2 (4%)		41 (6.4%)	
Clinical variables					
Diagnosis			χ^2^ = 0.06, p = .81		
Schizophrenia/non-affective FEP	77 (75%)	39 (78%)		412 (65%)	
Schizoaffective/affective FEP	26 (25%)	11 (22%)		172(27%)	
Other	0 (0%)	0 (0%)		52 (8%)	
Antipsychotic use			χ^2^ = 4.11, p = .13		χ^2^ = 17.5, p < 0.001
Currently using	86 (83%)	45 (90%)		447 (70%)	
Not currently using	9 (9%)	5 (10%)		76 (12%)	
Missing/not specified	8 (8%)	0 (0%)		113 (18%)	

^a^Statistics reported for differences between fBIRN and UCLA persistent psychotic illness samples.

^b^Statistics reported for for differences between MPEPP and other Samples.

### Statistical methods

#### Persistent psychotic illness sample

Analyses were completed in R-Version 1.0.153 using MASS, psych, pracma and tidyr packages. To assess the relationships between items on SAPS and SANS, we applied non-metric MDS to Euclidean distances estimated between items^19^. MDS reduces dimensionality by maintaining dissimilarity between items as relative distances in a new space with the fewest possible dimensions. Nonmetric MDS is ideal for ordinal measures^19^ and requires fewer dimensions because it does not assume the distance function is linear. Literature suggests n = 153 is sufficient to characterize SAPS and SANS items into 2, 3 or 4 dimensions, and the solution is largely insensitive to increasing sample size^50^ and stress measures are generally stable and reliable at this sample size^51^.

Consistent with prior work, and because we were interested in item inter-relation, we excluded items for which fewer than 10% of participants scored ≥ 2 and items with low communality with other items^19^. These exclusion criteria remove scale items which: (1) very few participants experience and thus are difficult to meaningfully identify relationships with other symptoms (low endorsement) and (2) have little in common with any other items on these scales, contributing little to the overall structure of symptoms (low communality). To determine items with low communality, we calculated squared multiple correlation coefficients and adjusted correlation metrics (multiple correlation coefficients can be inflated with this number of variables and sample size): an adjusted R^2^ and a more stringent index from Browne and colleagues^52^. In line with previous work^19^ items with Browne index < 0.15 were excluded from subsequent analyses (Table S1; Figs. 1C and 3A). To confirm that our exclusion criteria and choices about which items to include were not biasing our results and the conclusions we draw, we also ran MDS with different sets of items included and tested the congruence of our solutions. Here we also confirm whether our exclusion criteria increase goodness-of-fit and explained variance of our model.

We determined the optimal number of dimensions by quantifying stress across solutions with different dimensions. Stress is a goodness-of-fit metric of the difference between the observed similarity matrix and the estimated pairwise distance matrix. Furthermore, in order to determine whether our model performed significantly above chance, we permuted the raw scale data^53^, one item at a time and then calculated the correlations for that permuted item with all other unpermuted items. A permutation test was chosen because it does not rely on assumptions about the population distribution^53^ and is a superior method for determining robustness of MDS for a given dataset as compared to stress rule-of-thumbs^51^. We repeated this until all correlations were re-computed and applied non-metric MDS to the re-computed correlation matrix. This method creates a more informative null distribution of stress values than permuting across all columns at once^53^. We repeated 500 iterations and calculated the stress for each model in order to generate a null distribution for comparison with the experimental model. We then tested the underlying organization of symptoms by k-means clustering using the factoextra package and established methods for optimal cluster number selection based on gap statistics^54^ using the cluster package in R.

We also followed this up by running PCA, an independent dimensionality reduction method, applied just to hallucinations and delusion items of the SAPS, to determine whether an independent technique identified similar subdivisions of these items as visualized in our MDS (Supplementary Methods).

#### FEP sample

We repeated this MDS analysis in the independent sample of FEP patients. We only included items that met the communality and endorsement inclusion criteria for both persistent and first-episode samples to assess congruence across samples. We repeated MDS analysis for the persistent psychotic illness dataset, including items that met inclusion criteria for both datasets, and applied Procrustes transformation to this 2-dimensional solution. This uses rotations, translations and uniform scaling to reduce differences between dimension sets that are not driven by these data and will not affect the meaningful distances between items^53^. This transformation thus allowed us to calculate the congruence between solutions.

We further tested the relationships amongst symptoms in FEP patients with affective or non-affective psychosis. We ran non-metric MDS on both sub-samples and a Procrustes transformation to calculate the congruence between sub-samples.

## Supplementary Information

Supplementary Information.

## Data Availability

The Chronic Schizophrenia data are available here (https://openfmri.org/dataset/ds000030/) and here (http://www.schizconnect.org). Because the FEP participants did not provide informed consent to sharing their data, the FEP cohort data cannot be made publicly available. Those interested in exploring opportunities to examine this data are welcome to contact Dr Jai Shah for further details, including IRB guidance and processes.

## Code availability

Analysis code is available here: https://github.com/leahfleming/Symptom-organization.
