# Supplementary material for: Using dimensionality-reduction techniques to understand the organization of psychotic symptoms in persistent psychotic illness and first episode psychosis
Source: Sci Rep. 2023 Mar 24;13:4841. doi: 10.1038/s41598-023-31909-w (PMC10039017; doi:10.1038/s41598-023-31909-w)
Supplement: Supplementary file 1 — Supplementary Information. [file 41598_2023_31909_MOESM1_ESM.docx]

Supplemental Materials

**Supplementary Methods:**

**Participants**

*Persistent Psychotic Illness*

103 participants came from Phase II of the functional biomedical informative research network (fBIRN) data repository (Project Accession Number 2007-BDR-6UHZ1, accessed through Schizconnect website ([http://schizconnect.org](http://schizconnect.org/))). These data were collected from seven sites. Cross-site trainings were delivered by experienced raters and standardized with videotaped interviews[^25^](#_ENREF_25). Written informed consent was obtained from all participants. All had a diagnosis of schizophrenia or schizoaffective disorder, confirmed using the Structured Clinical Interview for DSM-IV (SCID-IV) (see[^26^](#_ENREF_26)^,^ [^27^](#_ENREF_27) for further information). Sample demographics are in Table 1.

Another 50 participants with a SCID-IV diagnosis of schizophrenia or schizoaffective disorder, were collected at University of California-Los Angeles[^28^](#_ENREF_28)^,^ [^29^](#_ENREF_29). These were obtained from the OpenfMRI database (<https://openfmri.org/dataset/ds000030/>), accession number ds000030. (see[^28^](#_ENREF_28) for further information). There were no significant demographic differences between these datasets (Table 1).

**Statistical Methods**

*PCA*

We confirmed our data were factorable based on a Kaiser-Meyer-Olkin measure of sampling adequacy value of 0.8 (recommended value>0.6)[^30^](#_ENREF_30) and a significant Bartlett’s test for sphericity (p<.0001). We centered and scaled these data and ran PCA on the correlation matrix. To improve interpretability, we applied Promax rotation to the loadings of components with eigenvalues>1 (selected according to “Kaiser rule”)[^13^](#_ENREF_13)^,^ [^14^](#_ENREF_14) to achieve a simple structure in the loadings.

Selecting eigenvalues>1 is prone to over-extraction, so it is possible the number of components optimal to explain this data is less than the number selected here. Therefore, we cannot claim that 5 components are optimal to explain hallucinations and delusions. However, this does not impact our ability to examine how the organization revealed by PCA compares to that derived via MDS and how the solutions compare across our two patient groups.

This type of rotation is ideal for identifying components which are likely highly related to one another since it is an oblique rotation. We confirmed that the oblique rotation was justified since most correlation coefficients between factors were >0.3 (Table S2a). This allowed us to quantify the relative relationship (correlation) amongst rotated components. Thus we could identify latent components that may underlie the variance across all reality distortion items; similar techniques have been applied in the past to delusions[^12^](#_ENREF_12)^,^ [^13^](#_ENREF_13) and hallucinations[^14^](#_ENREF_14) alone. Finally, we repeated PCA with the FEP sample, calculated congruence, and applied Promax rotation.

| **TABLE S1.** Communality estimates for all symptom items (rated at least 2 by $\geq10\% paticipants$. | | | | | | | | | | |
| --- | --- | --- | --- | --- | --- | --- | --- | --- | --- | --- |
|  |  | Yale Replication | | | | | McGill Replication | | | |
| *Symptoms* | | | $R^{2}$ | *Adjusted* $R^{2}$ | *Brown Index* | $R^{2}$ | | *Adjusted* $R^{2}$ | *Browne index* |  |
| SAPS Items  Auditory hallucinations  Voices commenting  Voices conversing  Somatic or tactile hallucinations  Olfactory hallucinations  Visual hallucinations  Persecutory delusions  Delusions of guilt or sin  Delusions of jealousy  Grandiose delusions  Religious delusions  Somatic delusions  Delusions of reference  Delusions of being controlled  Delusions of mind reading  Thought broadcasting  Thought insertion  Thought withdrawal  Clothing and appearance  Social and sexual behavior  Aggressive and agitated behavior  Repetitive and stereotyped behavior  Derailment  Tangentiality  Incoherence  Illogicality  Circumstanciality  Pressure of speech  Distractible speech  SANS Items  Unchanging facial expression  Decreased spontaneous movements  Paucity of expressive gestures  Poor eye contact  Affective non-responsivity  Inappropriate affect  Lack of vocal inflections  Poverty of speech  Poverty of content of speech  Blocking  Increased latency of response  Grooming and hygiene  Impersistence at work or school  Physical anergia  Recreational interests and activities  Sexual activity  Ability to feel intimacy and closeness  Relationships with friends and peers  Social inattentiveness  Inattentiveness during mental status testing | | | 0.73  0.67  0.68  0.57  0.51  0.53  0.53  0.52  0.52  0.58  0.57  0.57  0.64  0.62  0.75  0.69  0.74  0.73  0.54  0.44  0.41  0.84  0.74  0.65  0.70  0.53  0.81  0.76  0.87  0.66  0.79  0.74  0.64  0.43  0.53  0.42  0.59  0.56  0.62  0.57  0.56  0.58  0.66  0.57  0.40 | 0.61  0.54  0.55  0.39  0.31  0.33  0.34  0.33  0.32  0.40  0.40  0.39  0.49  0.47  0.64  0.56  0.64  0.61  0.35  0.22  0.18  0.77  0.64  0.51  0.57  0.34  0.73  0.67  0.82  0.51  0.70  0.64  0.49  0.20  0.34  0.18  0.43  0.38  0.47  0.40  0.38  0.40  0.53  0.39  0.16 | 0.47  0.39  0.40  0.23  0.15  0.18  0.18  0.17  0.17  0.24  0.24  0.23  0.33  0.31  0.51  0.42  0.51  0.47  0.19  0.08*  0.05*  0.68  0.50  0.36  0.43  0.18  0.62  0.54  0.74  0.36  0.58  0.50  0.33  0.07*  0.18  0.06*  0.27  0.22  0.31  0.23  0.22  0.24  0.37  0.23  0.04* | 0.62  0.61  0.41  0.40  0.25  0.19  0.17  0.45  0.34  0.37  0.22  0.31  0.40  0.32  0.35  0.22  0.30  0.28  0.16  0.59  0.60  0.46  0.40  0.44  0.44  0.28  0.72  0.72  0.79  0.50  0.59  0.31  0.68  0.59  0.38  0.32  0.55  0.28  0.38  0.51  0.58  0.23  0.44  0.51  0.39  0.16 | | 0.60  0.58  0.36  0.36  0.20  0.12  0.10  0.40  0.29  0.32  0.16  0.26  0.35  0.26  0.30  0.16  0.25  0.22  0.10  0.55  0.57  0.42  0.36  0.39  0.40  0.22  0.70  0.70  0.78  0.46  0.56  0.25  0.65  0.56  0.33  0.26  0.52  0.22  0.33  0.47  0.54  0.17  0.40  0.47  0.34  0.09 | 0.56  0.55  0.32  0.31  0.15  0.08*  0.06*  0.36  0.24  0.28  0.11*  0.21  0.31  0.22  0.25  0.11*  0.20  0.17  0.05*  0.52  0.54  0.37  0.31  0.35  0.35  0.17  0.68  0.68  0.76  0.41  0.52  0.20  0.62  0.52  0.28  0.21  0.48  0.17  0.28  0.43  0.51  0.12*  0.36  0.43  0.29  0.05* |  |

**TABLE S2. Correlation coefficients (absolute value) between first five principal components after Promax rotation.**

1. *Persistent psychotic illness sample*

|  | PC1 | PC2 | PC3 | PC4 | PC5 |
| --- | --- | --- | --- | --- | --- |
| PC1 | 1.0 |  |  |  |  |
| PC2 | .55 | 1.0 |  |  |  |
| PC3 | .15 | .12 | 1.0 |  |  |
| PC4 | .29 | .32 | .15 | 1.0 |  |
| PC5 | .47 | .45 | .14 | .31 | 1.0 |

1. *McGill FEP replication sample*

|  | PC1 | PC2 | PC3 | PC4 | PC5 |
| --- | --- | --- | --- | --- | --- |
| PC1 | 1.0 |  |  |  |  |
| PC2 | .36 | 1.0 |  |  |  |
| PC3 | .22 | .18 | 1.0 |  |  |
| PC4 | .27 | .31 | .19 | 1.0 |  |
| PC5 | .26 | .20 | .08 | .17 | 1.0 |

**Figure S1. MDS solution with only SAPS items included from persistent psychotic illness sample.**

*CON=voices conversing, COM=voices commenting, SOM_H=somatic hallucinations, VIS=visual hallucinations, OLF=olfactory hallucinations, SOM_D=somatic delusions, GRA=grandiose delusions, REL=religious delusions, PERS=persecutory delusions, REF=delusions of reference, DCO=delusions of being controlled, GLT=delusions of guilt/sin, JEAL=delusions of jealousy, DMR=delusions of mind reading, SCL=social and sexual behavior, TAN=tangentiality, TBR=thought broadcasting, DER=derailment, TWD=thought withdrawal, INC=incoherence, ILL=illogicality, CIR=circumstantiality, PRE=pressure of speech, DST=distractible speech, TIN=thought insertion, GRM=grooming and hygiene, EXP=unchanging facial expression, SPO=less spontaneous activity, GES=paucity of gestures, EYE=poor eye contact, ANR=affective non-responsiveness, VIN=low vocal inflection, PSP=poverty of speech, BLK=blocking, IWS=impersistence at work, PAN=physical anergia, SEX=sexual activity, SOC=relationships with friends, INT=Inability to feel intimacy.*


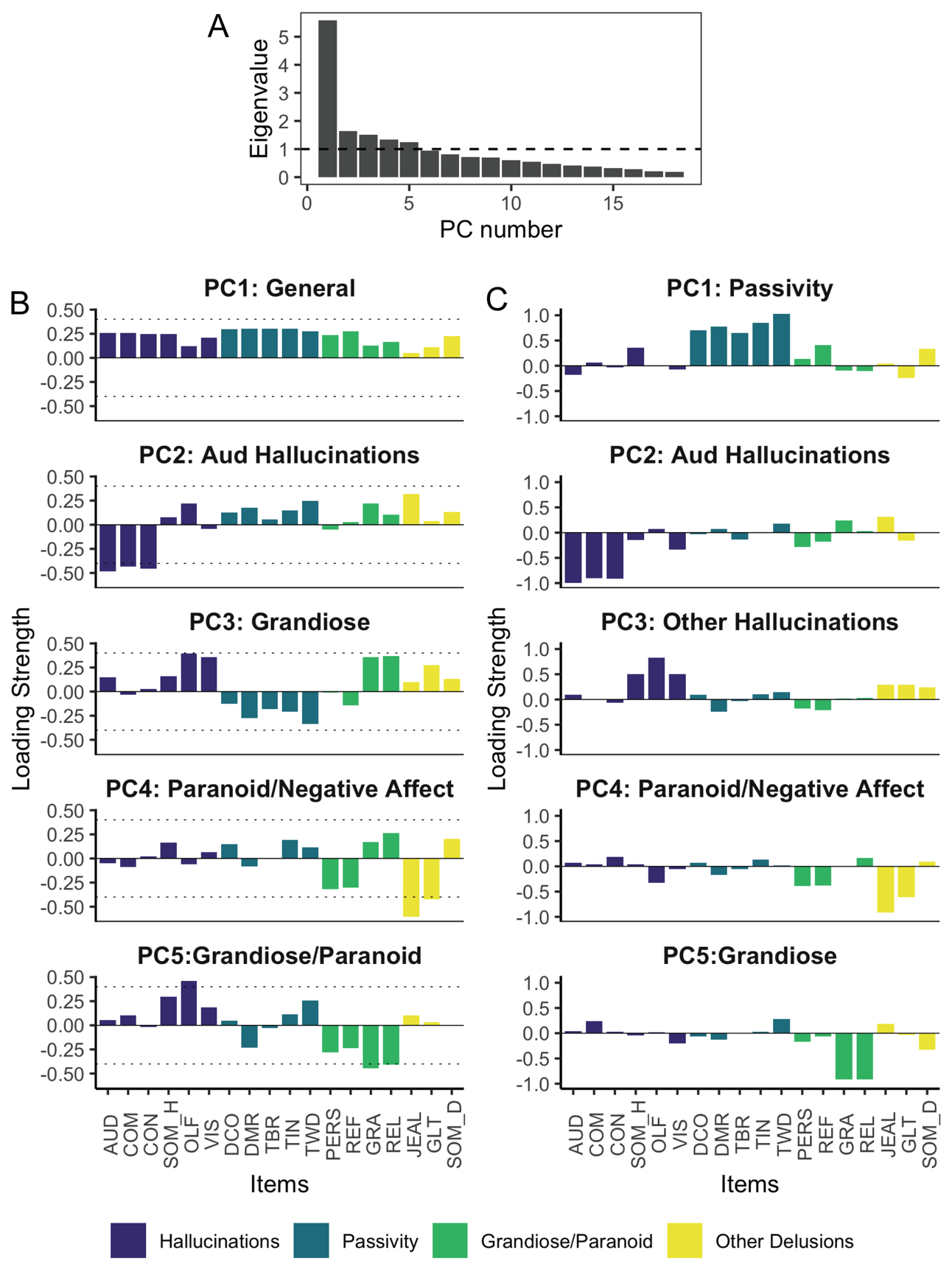


**Figure S2. Principal component analysis results for psychotic illness sample.** ^A^ Scree plot of eigenvalues across principal components for schizophrenia/schizoaffective disorder patients. Dashed, horizontal line depicts cutoff eigenvalue of 1. ^B^ Loadings of delusion and hallucination items from SAPS onto first 5 PCs, with color-coding to differentiate subgroups of symptoms previously identified in the literature. ^C^ Loadings for all items after Promax rotation. *CON=voices conversing, COM=voices commenting, SOM_H=somatic hallucinations, VIS=visual hallucinations, OLF=olfactory hallucinations, SOM_D=somatic delusions, GRA=grandiose delusions, REL=religious delusions, PERS=persecutory delusions, REF=delusions of reference, DCO=delusions of being controlled, GLT=delusions of guilt/sin, JEAL=delusions of jealousy, DMR=delusions of mind reading, SCL=social and sexual behavior, TAN=tangentiality, TBR=thought broadcasting, DER=derailment, TWD=thought withdrawal, INC=incoherence, ILL=illogicality, CIR=circumstantiality, PRE=pressure of speech, DST=distractible speech, TIN=thought insertion, GRM=grooming and hygiene, EXP=unchanging facial expression, SPO=less spontaneous activity, GES=paucity of gestures, EYE=poor eye contact, ANR=affective non-responsiveness, VIN=low vocal inflection, PSP=poverty of speech, BLK=blocking, IWS=impersistence at work, PAN=physical anergia, SEX=sexual activity, SOC=relationships with friends, INT=Inability to feel intimacy.*


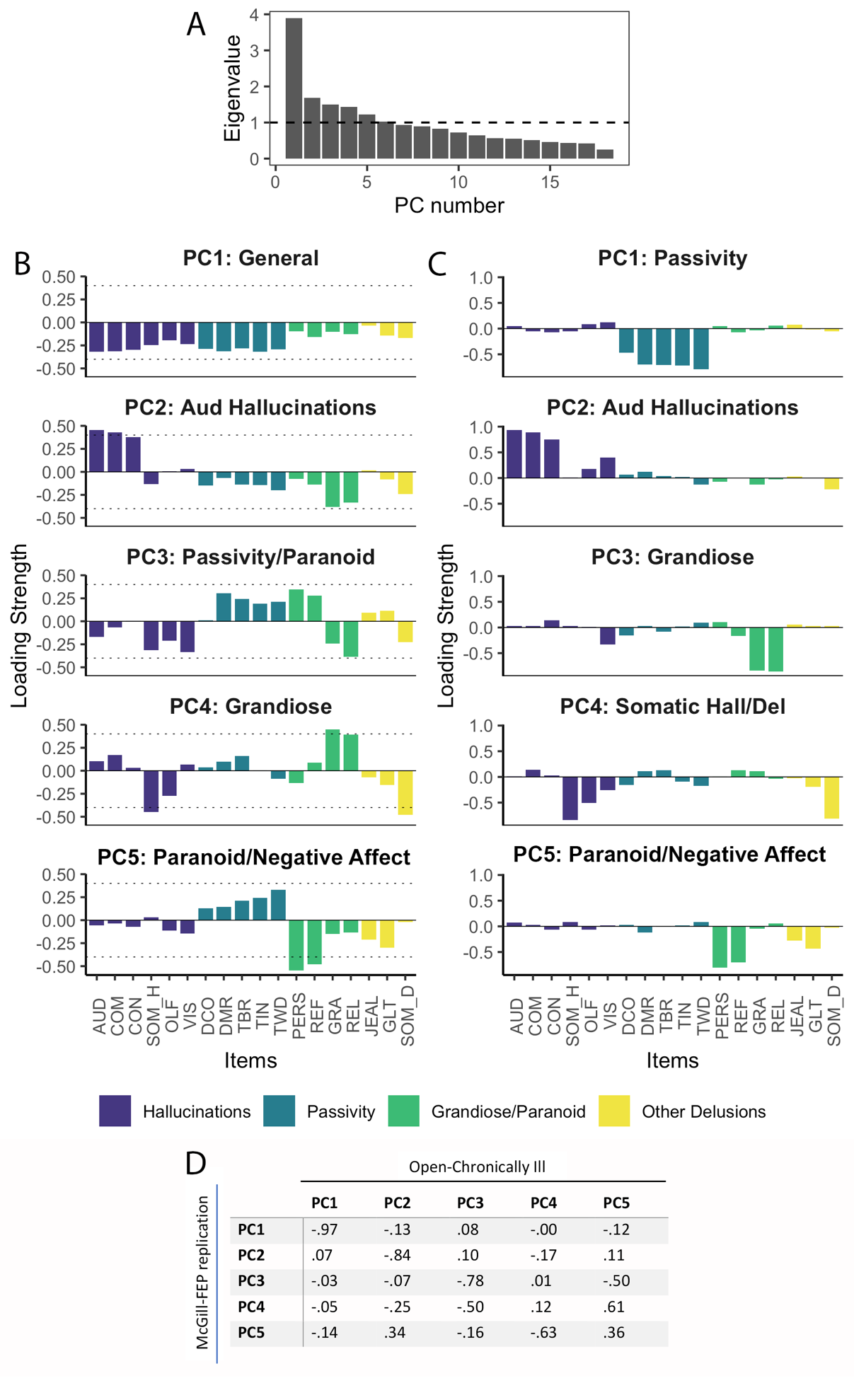


**Figure S3. Replication of principal component analysis results in FEP sample.**

^A^ Scree plot of eigenvalues across principal components for FEP patients. Dashed, horizontal line depicts cutoff eigenvalue of 1. ^B^ Loadings of delusion and hallucination items from SAPS onto first 5 PCs, with color-coding to differentiate subgroups of symptoms previously identified in the literature. ^C^ Loadings for all items after Promax rotation. ^D^ Congruence coefficients across first five PCs between the persistent psychotic illness and FEP samples. Note, components are organized in order of variance explained, not content of the components, so the 4^th^ and 5^th^ components in the persistent psychotic illness sample correspond most to the 5^th^ and 4^th^ of the FEP sample respectively. *CON=voices conversing, COM=voices commenting, SOM_H=somatic hallucinations, VIS=visual hallucinations, OLF=olfactory hallucinations, SOM_D=somatic delusions, GRA=grandiose delusions, REL=religious delusions, PERS=persecutory delusions, REF=delusions of reference, DCO=delusions of being controlled, GLT=delusions of guilt/sin, JEAL=delusions of jealousy, DMR=delusions of mind reading, SCL=social and sexual behavior, TAN=tangentiality, TBR=thought broadcasting, DER=derailment, TWD=thought withdrawal, INC=incoherence, ILL=illogicality, CIR=circumstantiality, PRE=pressure of speech, DST=distractible speech, TIN=thought insertion, GRM=grooming and hygiene, EXP=unchanging facial expression, SPO=less spontaneous activity, GES=paucity of gestures, EYE=poor eye contact, ANR=affective non-responsiveness, VIN=low vocal inflection, PSP=poverty of speech, BLK=blocking, IWS=impersistence at work, PAN=physical anergia, SEX=sexual activity, SOC=relationships with friends, INT=Inability to feel intimacy.*
